# Low-Income, Older African Americans’ Engagement in and Perceptions of a Smartphone-Based Ecological Momentary Assessment Study of Physical Activity and Sedentary Behavior

**DOI:** 10.1093/geroni/igab056

**Published:** 2021-12-23

**Authors:** Laurie Kennedy-Malone, Derek J Hevel, Kourtney B Sappenfield, Heidi Scheer, Christine Zecca, Jaclyn P Maher

**Affiliations:** 1 School of Nursing, University of North Carolina at Greensboro, Greensboro, North Carolina, USA; 2 Department of Kinesiology, University of North Carolina at Greensboro, Greensboro, North Carolina, USA; 3 Duke Clinical Research Institute, Durham, North Carolina, USA; 4 Alzheimer’s Association of Western Carolina Chapter, Charlotte, North Carolina, USA

**Keywords:** Accelerometer, Exercise/physical activity, Race, Recruitment, Technology

## Abstract

**Background and Objectives:**

Smartphone-based ecological momentary assessment (EMA) methods can provide novel insights into modeling and predicting activity-related behaviors, yet many racial and ethnic minority groups report barriers to participating in mobile health research. We aim to (a) report on strategies used to successfully recruit and retain minority older adults in a smartphone-based physical activity and sedentary behavior EMA study and (b) report on participants’ perceptions of study acceptability.

**Research Design and Methods:**

Researchers partnered with trusted individuals and community organizations serving older adults to facilitate recruitment for an 8-day EMA study of minority older adults’ physical activity and sedentary behavior. Additional strategies such as having experienced, culturally competent team members and available technical support were employed to further recruitment and retention efforts. A poststudy questionnaire assessed participants’ perceptions of study acceptability.

**Results:**

In total, 123 minority older adults were recruited, 102 met inclusion criteria, 91 completed the study, and 89 completed the poststudy questionnaire. The sample consisted of predominantly low-income African American women with an average age of 70 years. Responses to open-ended questions revealed that the most enjoyable aspects of study participation were the ability to learn more about themselves, contribute to science and/or their community, engage in a new activity, and receive financial compensation. Participants reported the least enjoyable aspects of the study included the frequency of EMA questionnaires, apprehension of missing EMA questionnaires, carrying the smartphone, and difficulty wearing the accelerometer. Most participants (97%) expressed interest in being contacted for future studies.

**Discussion and Implications:**

Low-income, older African Americans reported positive perceptions of a smartphone-based EMA study of physical activity and sedentary behavior. Findings suggest that applying demonstrated strategies to engage this population in technology-based health research can enhance recruitment and retention efforts; however, it is unclear which strategies are most effective in reducing participation barriers.

**Translational Significance:** When leveraging familiar community gatekeepers and using demonstrated strategies known to enhance recruitment among minority older adults, the targeted sample of low-income, older African Americans was achieved within 5 months in a study aimed at understanding physical activity and sedentary behavior using ecological momentary assessment (EMA). Understanding the factors that motivated participants to enroll and complete an EMA health-related research study can inform recruitment for decentralized research among low-income, older African Americans.

## Background and Objectives

Physical inactivity and excessive sedentary behavior are major public health threats, especially for older adults. Recent estimates suggest that between half and three quarters of older adults do not meet physical activity guidelines (Keadle et al., 2016). Furthermore, the average older adult sits for three fourths of their waking hours, equivalent to 12 h of sitting in a 16-h day (Copeland et al., 2015; Diaz et al., 2017). Additionally, there are racial disparities among older adults regarding physical activity and sedentary time. For instance, African American older adults engage in less physical activity and have more sedentary behavior compared to their White counterparts (Evenson et al., 2014).

An incomplete understanding of the factors contributing to physical activity and sedentary behavior can perpetuate health disparities in movement-related behaviors (Lachman et al., 2018). Ecological momentary assessment (EMA) methods can provide novel insights into the modeling and prediction of health behaviors that vary across time and contexts, such as physical activity or sedentary behavior, due to its intensive assessments designed to occur as individuals go about their daily lives (Dunton, 2017; Stone & Shiffman, 1994). Smartphone-based EMA is gaining popularity in physical activity research (Bruening et al., 2016; Dunton et al., 2012; Knell et al., 2017), yet there has been little research, comparatively, that uses EMA to capture and understand activity-related behaviors among older minority adults (Maher et al., 2018).

Underrepresentation of minority older adults in research further contributes to health disparities related to physical activity and excessive sedentary behavior. Minority groups report barriers to participating in health-related research such as skepticism of the research or distrust of the researchers, lack of interest in the study, privacy or surveillance concerns, and lack of transportation to the study site (James & Harville, 2017; Liljas et al., 2017; Shapiro et al., 2017). When seeking representation from minority older adults from community sites, it is essential to be aware of potential underlying community-based barriers such as perceived exploitation of a vulnerable population, lack of knowledge of the benefit from health-related research, and the apparent reluctance of the researchers to establish relationships with the community members (George et al., 2014; Kammerer et al., 2019; Shapiro et al., 2017). However, because of the lack of EMA studies designed to study physical activity and sedentary behavior among racial and ethnic minority populations, it is unclear if such strategies would be effective in recruiting and retaining minority older adults to a smartphone-based EMA study of physical activity and sedentary behavior and what perceptions of such a study would be in this population.

The purpose of this article is twofold. First, we aim to report on strategies used to successfully recruit and retain minority older adults in an 8-day smartphone-based physical activity and sedentary behavior EMA study. Second, we aim to report on participants’ perceptions of study acceptability.

## Research Design and Methods

### Project ABLE

From February to July 2018, minority older adults were recruited to participate in Project ABLE (Adults Behaviors in Living Environments), which was an 8-day repeated-measures observational study designed to determine psychosocial antecedents and consequences of minority older adults’ physical activity and sedentary behavior. Inclusion criteria for the study were age 60 years or older, self-reported being able to speak or read English fluently, identifying as at least one racial or ethnic minority group, and living in Guilford County, NC. Exclusion criteria included if they self-reported functional limitations that prevented standing or walking on their own or seeing or utilizing the smartphone or scored ≤31 on the Modified Telephone Interview for Cognitive Status designed to detect mild cognitive impairment (Knopman et al., 2010).

### Gaining Entrée to Potential Participants

To access community-dwelling older African Americans, researchers partnered with existing community organizations and trusted individuals to introduce the potential participants to the research team and the study objectives. The research team connected with colleagues from a group of university gerontology faculty and community affiliates interested in promoting age-related research, education, and outreach at a networking campus event. Through connections established at this meeting, the research team could converse with leaders of the county’s older adult resource center, an organization that facilitates health-related services such as the community nutrition programs. As a result, the research team was able to recruit from and train participants at several congregate meal site locations. Meal site managers also facilitated introductions of the research team with potential participants, assuring to the older adults the researchers’ interest in their well-being was genuine. The research team also had access to wellness centers within subsidized housing independent living centers in central North Carolina. The research team approached the wellness center coordinator to determine the feasibility of conducting the study at each of the sites. Serving as a trusted conduit to the residents of the independent living centers, the wellness coordinator assisted with the posting of recruitment materials, allowed the research team to make recruitment announcements following residents’ appointments, and assisted the recruited participants with any initial questions outside of scheduled study appointments with the research team. Additionally, participants were encouraged to share their experiences to spark interest in others who might volunteer for the study.

### Strategies to Facilitate Recruitment and Retention

In addition to working with gatekeepers at trusted community organizations, several strategies were employed to recruit and retain minority older adults successfully in this study. First, our research team, including those involved in recruiting and training participants, had extensive clinical experience with practical knowledge and tacit experience with minority older adults. This knowledge and experience aided communication that was sensitive to health literacy for effectively communicating study procedures. Second, our approach to recruitment included culturally sensitive recruitment materials such as study flyers adapted to feature older African Americans. Third, during the recruitment process, the research team displayed personal interest with the potential participants, listening and learning about their lives, well-being, and concerns. As part of this process, when applicable, the research team connected these older adults with community resources (e.g., nonprofits designated to support seniors’ physical and emotional needs), which was especially relevant in subsidized housing independent living centers. Fourth, our team coordinated with congregate meal sites and subsidized housing locations to deliver participant training at these community locations. Finally, we had a technical support line that participants could call during the study to report issues and receive assistance with study equipment. This phone was monitored during business hours as well as evenings and weekends to provide continual technical support. In summary, these strategies were designed to enhance recruitment and retention efforts by (a) establishing trust and rapport between community members and the research staff, (b) effectively communicating the individual- and community-level benefits from this health-related research, and (c) minimizing logistical barriers to participating.

### Procedures

On Day 1, participants were trained on the study equipment by attending an in-person, one-on-one, or small group introductory session. The introductory session was held at the same locations from where individuals were recruited either in the wellness centers of the community living sites or at the congregate meal sites. Participants recruited through word of mouth were given the option of attending training sessions at these recruitment sites or their own homes. Utilizing these locations fostered another form of relationship development due to participant familiarity and convenience of place/locale. The university’s Institutional Review Board approved all study procedures, and all participants provided written informed consent before being trained on the study equipment.

All participants were loaned a MotoG4 (Motorola, Inc.) smartphone and trained on how to use the smartphone to answer EMA questionnaires as part of the study. Each phone was preloaded with the smartphone application MovisensXS, which was used to deliver the EMA questionnaires. Participants received six EMA questionnaires per day randomly between 8:00 a.m. and 8:00 p.m. Each EMA questionnaire contained up to 20 items assessing current activity, current social and physical context, affective state, and motivation for engaging in physical activity and reducing sedentary time over the next 2 h. When an EMA questionnaire was delivered, participants were notified with an auditory signal and/or vibration (depending on the volume settings participants had set). Participants had 15 min to respond to the EMA questionnaire. If participants did not respond to the initial notification, they received reminder notifications at 5-min intervals until they reached the 15-min threshold. After 15 min, the EMA questionnaire expired, and participants could no longer respond to that EMA questionnaire and were counted as one that was missed. Each EMA questionnaire was expected to take 2–3 min to complete, for a total of 12–18 min per day.

Also, during the Day 1 introductory session, participants were trained to wear an ActivPAL activity monitor on their anterior thigh 3 inches above the knee continuously for the duration of the study. Participants used Hypafix adhesive bandages to adhere the monitor to their thigh during the introductory session. The research staff member conducting the introductory session was available to help participants in this process if assistance was requested. The activity monitor was sealed in polymer tubing to waterproof the monitor to allow participants to wear the monitor during water-based activities (e.g., bathing). Before leaving the introductory session, participants completed a paper-and-pencil questionnaire to assess demographic information.

Participants were given the research team’s telephone number and email address and encouraged to make contact if concerns arose. The study phone did not have an active data plan so participants could not make or receive calls on it. Therefore, participants had to use another device to contact the study team if needed. Between Days 2 and 4 of the study, the research team contacted participants via their preferred method of communication to check in and inquire about any study-related issues.

After completing the 8-day study, participants were scheduled to attend a poststudy appointment to return study equipment, complete a final questionnaire requesting feedback about their experience in the study, and receive remuneration. Participants could receive up to $75 for completing the study ($10 for attending the introductory session, $50 for completing the 8-day study, and $15 for answering at least 75% of the EMA questionnaires on the smartphone). Each participant was given an individualized report from the activity monitor about their physical activity and sedentary behavior after the study.

### Measures

Demographic information assessed included age, sex, race, ethnicity, annual household income, measures of height and weight taken in duplicate by a trained research assistant, and information about their current residence. Self-reported physical activity was assessed using the International Physical Activity Questionnaire (Craig et al., 2003). Self-reported sedentary behavior was assessed using a nine-item scale, modified from existing validated measures of sedentary behavior (Gardiner et al., 2011; Visser & Koster, 2013). The assessment used in this study assessed domain-specific sedentary activities including time spent watching TV, using the computer, reading, socializing with friends, in transit, completing hobbies, doing paperwork, eating, or any other activities. Separate sets of items assessed weekday and weekend domain-specific sedentary behavior.

To assess study acceptability of participating in an EMA study addressing physical activity and sedentary behavior, participants self-completed a paper-and-pencil poststudy questionnaire after the 8-day study. Six items asked participants to reflect on different aspects of participating in the study including (a) the extent to which the daily demands of the study were reasonable, (b) participants’ thoughts about the length of the EMA questionnaires, (c) the experience of wearing the activity monitor every day, (d) the appropriateness of the compensation, (e) participants’ willingness to participate in future research, and (f) participants’ willingness to participate in another data collection with identical study procedures described in this article after a 6-month break. The full questions and associated responses are given in Table 1. Two open-ended questions asked participants to identify the most and least enjoyable aspect of their participation in the study.

**Table 1.** Poststudy Questionnaire Responses

Item and response options	n	%
“The daily demands of this study were reasonable?”		
Strongly disagree	1	1.12
Disagree	3	3.37
Neither agree nor disagree	15	16.85
Agree	57	64.04
Strongly agree	12	13.48
Missing	1	1.12
“How did you feel about the length of the questionnaires?”		
Way too long	3	3.37
A little long	7	7.87
Reasonable length	75	84.27
A little short	3	3.37
Way too short	1	1.12
“Wearing the activity monitor everyday was a nuisance.”		
Strongly disagree	20	22.47
Disagree	50	56.18
Neither agree nor disagree	9	10.11
Agree	8	8.99
Strongly agree	2	2.24
“Overall, I found the demands of participating in this study to be reasonable for the compensation.”		
Strongly disagree	2	2.24
Disagree	2	2.24
Neither agree nor disagree	4	4.49
Agree	64	71.91
Strongly agree	15	16.85
Missing	2	2.24
“May we contact you about opportunities to participate in future research studies?”		
No	2	2.24
Yes	86	96.63
Missing	1	1.12
“If you were asked to complete additional 8-day periods of data collection with a 6-month break in between each data period, would you?”		
No	6	6.74
Yes	60	67.42
Maybe	23	25.84

### Data Analysis

Frequencies were calculated for survey items. Categories were identified in open-ended responses. Chi-squared tests and *t*-tests were conducted, where applicable, to determine differences in enrollment rates and study acceptability responses by recruitment location and smartphone ownership status. Two investigators (D. J. Hevel and K. B. Sappenfield) independently analyzed raw open-ended responses then met to discuss and resolve discrepancies in the categorization by consensus.

## Results

The targeted sample size for the study was 100 minority older adults with the intention of 85 completing the study. In total, 123 minority older adults, primarily African American, were recruited, 102 met inclusion criteria, 91 enrolled and completed the study, and 89 completed poststudy questionnaires. Twenty-one of those initially interested in participating in the study did not pass the Modified Telephone Interview Cognitive Status. Eleven of the participants who met the inclusion criteria chose not to enroll (*n* = 6, no longer interested in study; *n* = 3, personal/family medical issue; *n* = 1, too busy; *n* = 1, unknown). All participants who enrolled in the study completed the study, serving as an indicator of strong study retention. Of those who completed the study, about half (52.7%) were recruited from the subsidized housing independent living centers where the wellness centers are located, 25.3% were from congregate meal sites, and the remaining participants were recruited through word of mouth. Because two participants did not complete the poststudy questionnaire, the final analytic sample was 89 minority older adults.

Older minority adult participants (*M* = 70.02 years, *SD* = 5.71) were mostly female (77.5%) and identified as Black or African American (89.0%), two or more races (7.7%), other (2.2%), or Asian (1.1%). Based on calculated body mass index (BMI) from height and weight measurements, most were overweight (28.6%) or obese (57.1%) with an average BMI of 31.51 (*SD* = 6.61). Participants reported an annual household income of less than $4,999 (25.3%) or $5,000–19,999 (45.1%), while some did not report (11.0%), and the majority reported living alone (72.5%). Regarding health, 5.5% rated their overall health as excellent, 24.2% rated as very good, 45.1% rated as good, 23.1% rated as fair, and 1.1% rated as poor (one participant did not respond). Among the sample, the most commonly diagnosed chronic conditions reported were high blood pressure (82.4%), high cholesterol (53.8%), and type II diabetes (28.6%). On a typical weekday, participants self-reported spending an average of 14.58 h (*SD* = 4.28) of sedentary time and, on a typical weekend day, spending an average of 13.75 h (*SD* = 4.42) of sedentary time. Additionally, participants self-reported spending an average of 55.07 min (*SD* = 59.98) of moderate- to vigorous-intensity physical activity per day. Based on ActivPAL data, participants took 4,934 steps per day, on average (*SD* = 3,346). Participants, on average, completed 92% of the EMA prompts (Mean_# of occasions_ = 43, Median_# of occasions_ = 43, *SD*_# of occasions_ = 5.75, Range_# of occasions_ = 10–48). On most occasions, it took participants less than 3 min to complete the EMA questionnaire (*M* = 2.6 min, *SD* = 1.8 min).

Comparisons across recruitment sites indicated that there were differences in age (*F*(2, 87) = 9.02, *p <* .01), sex (*F*(2, 88) = 11.21, *p <* .01), income (*F*(2, 78) = 4.07, *p =* .02), and average daily steps (*F*(2, 88) = 7.60, *p <* .01) across recruitment strategies. Participants who were recruited from congregate meal sites tended to be older (*M* = 74.30) than those recruited from wellness clinics (*M* = 68.59) and by word of mouth (*M* = 69.02). Participants who were recruited from wellness clinics tended to include a greater proportion of men (*M* = 0.39) compared to those recruited from congregate meal sites (*M* = 0.04) or by word of mouth (*M* = 0.00). Participants who were recruited via word of mouth tended to report a higher household income (*M* = 1.71) and engage in more steps per day (*M* = 6,851.65) compared to those recruited from congregate meal sites (income: *M* = 0.85; steps: *M* = 5,114.84) and wellness clinics (income: *M* = 0.89; steps: *M* = 4,250.11). There were no significant differences in BMI or self-reported physical activity or sedentary behavior across recruitment sites.

Poststudy questionnaires revealed that the daily demands of this study were reasonable (64% agree; 13% strongly agree). Most participants (84%) found the length of the EMA questionnaires to be reasonable, 56% disagreed, and 22% strongly disagreed that wearing the accelerometer every day was a nuisance. Participants also found that the study demands were reasonable for the incentive provided (71% agreed; 17% strongly agreed). Furthermore, 97% of participants responded “yes” to be contacted for future research, and 67% responded “yes” to be willing to complete another data collection period with identical study procedures after a 6-month break. Complete responses are given in Table 1. Across recruitment sites, only the extent to which participants perceived the daily time demands of the study as reasonable differed across recruitment sites, *F*(2, 83) = 3.32, *p =* .04. On a 1 (*strongly disagree*) to 5 (*strongly agree*) response scale, participants recruited by word of mouth indicated significantly higher levels of agreement (*M* = 4.10) compared to those recruited from wellness clinics (*M* = 3.90), and those recruited from wellness clinics indicated significantly higher levels of agreement than those recruited from congregate meal sites (*M* = 3.55).

Responses to the open-ended question about the most enjoyable aspects of the study most commonly included engaging with study procedures (e.g., answering questions on the smartphone; 36%), the opportunity to learn about themselves (28%), contributing to science/their community (10% of participants), and receiving financial compensation (6% of participants). Additionally, 8% of participants did not provide the most enjoyable aspect of the study. Participants’ responses to the open-ended question about the least enjoyable aspects of the study most commonly included apprehension related to the EMA prompts (18%), the frequency of the EMA prompts (11%), difficulty with wearing the activity monitor (9%), and carrying the smartphone (3%). Additionally, 17% of participants did not respond to the least enjoyable aspect of the study.

## Discussion and Implications

This study highlights potentially effective recruitment and retention strategies for technology-based health behavior research among an understudied population. Additionally, the findings from this study suggest that low-income, older African Americans are willing and able to participate in EMA studies pertaining to physical activity and sedentary behavior. A positive experience participating in research studies is likely for low-income, older African Americans if researchers are willing to actively engage with and recruit from community-based organizations willing to promote and facilitate the study.

Previous research has identified successful strategies for recruiting older African Americans to participate in health-related research. One of the most effective strategies in recruiting this population is establishing trust between the population of interest and the research staff (Kammerer et al., 2019; Northridge et al., 2017; Shapiro et al., 2017). To establish trust, our research team utilized established academic–community partner sites that minority older adults frequent and trusted individuals within those sites to facilitate recruitment (Kammerer et al., 2019; Liljas et al., 2017; Ramsay et al., 2020; Simning et al., 2015). For researchers who do not have academic practice networks that focus on community resources for older adults, establishing such a partnership independently may facilitate opportunities for the recruitment of older adults for research studies (Mahoney et al., 2020). However, it is important to recognize that these partnerships take time to cultivate, so researchers must leave adequate time to initiate and develop these relationships prior to the commencement of research. Additionally, engaging community partners in research design can help to facilitate buy-in and allow for meaningful outcomes for both parties (Hudson et al., 2020; Mahoney et al., 2020). Practical content (e.g., presenting study findings, wellness strategies) and/or financial compensation, if available, to community organizations may be necessary to incentivize community organizations’ participation in a given project. Other strategies used to facilitate recruitment included employing staff that had previous clinical experience working with the intended population and developing recruitment materials that included culturally relevant images and considered health literacy, which is also consistent with previous research involving vulnerable, understudied populations (McHenry et al., 2015; Northridge et al., 2017).

Our research team also attempted to remove logistical barriers related to the location of study appointments and phone ownership to enhance participants recruitment and retention further. Previous research suggests that by eliminating transportation barriers and the need to navigate an unfamiliar setting (e.g., university campus), older adults from understudied populations are more likely to participate and complete health-related research (Liljas et al., 2017). Conducting appointments in locations viewed as familiar and safe by participants likely created another form of trust between members of the research team and study participants (Simning et al., 2015). Furthermore, by not requiring participants to own a smartphone, logistical barriers for low-income participants were potentially reduced. As a result, this is one of the first studies to recruit a primarily low-income sample of older African Americans to mobile technology physical activity research.

Retention in the present study was high. All participants who consented to participate in the study completed the study. Additionally, more than two thirds and one quarter of participants indicated that they would participate or would consider participating in an additional data collection period employing identical study procedures, respectively. Remuneration is typically considered an effective strategy to entice individuals to participate and stay enrolled in EMA research, especially when financial compensation is prorated based on the number of EMA prompts answered throughout the study (Christensen et al., 2003; Musthag et al., 2011). However, remuneration is often not considered the main reason for participating in research studies cited by ethnic and racial minorities (Ejiogu et al., 2011; George et al., 2014). Results from the present study tend to support the idea that compensation may not sway older African American adults to participate or stay enrolled in a health-related EMA study, as only 6% of the participants who completed the study indicated incentives were the most enjoyable aspect of the study despite most of the sample having an annual income below $20,000, although previous research suggests that older participants do appreciate that their time commitment is recognized by receiving monetary incentives (McHenry et al., 2015; Northridge et al., 2017) and in the present study, nearly 90% of participants reported that the compensation was appropriate for the demands of the study. In addition to financial compensation, this study provided those participants who completed the study with reports on their activity-related behaviors based on data from their activity monitor. Work by McHenry et al. (2015) suggests that reports or feedback may create a tangible personal benefit of the research, enhancing recruitment and retention efforts.

However, there was no formal assessment of whether our attempts to reduce or eliminate recruitment- or retention-related barriers reduced barriers typically cited by this population. Future technology-based health research among older African Americans should address which strategies employed are particularly impactful in reducing barriers to participation among this population. Better understanding of motives related to recruitment and retention may also have implications for the acceptability of such research among vulnerable, understudied populations.

Based on descriptive findings from the present study, the overall design and specific procedures employed were acceptable among older African Americans. First, participants complied with the study procedures at a high rate. Overall compliance with the EMA protocol in the present study was nearly identical to previous EMA research among mostly older White adults, with participants answering approximately 92% of the EMA prompts (Maher et al., 2018). Additionally, compliance in the present study was similar to another EMA study of older African Americans’ activities of daily living and stress (Fritz et al., 2017), all of which suggests that older African Americans are willing to participate in technology-based, intensive health-related research and, when adequately trained, are able to complete it successfully. Second, older African Americans indicated that the activity monitor was not a nuisance. Despite privacy or surveillance concerns frequently cited as a barrier to participation among minority groups (James & Harville, 2017; Liljas et al., 2017; Shapiro et al., 2017), participants in this study appeared pleased with wearing the activity monitor. This behavior may speak to the recruitment and training activities in which participants were shown the activity monitor, informed of what information the activity monitor (e.g., acceleration) did and did not record (e.g., location), and were able to ask questions about the monitor. It should be noted, however, that the requirement of wearing a device taped to the body was a barrier for a few participants who indicated interest but who later declined. Finally, nearly all participants indicated they would like to be contacted about future research opportunities, and more than two thirds indicated that they would participate in the present study again. It is important to note that only 6.7% of participants definitively said they would not participate in the present study again. One quarter of participants (25.8%) responded “maybe” to participating in the present study again and the most common themes influencing the likelihood of participation for those who indicated “maybe” were timing/flexibility in scheduling and compensation. In summary, our data suggest that the integration of smartphone-based EMA with accelerometry is a feasible and acceptable approach to studying activity-related behaviors and their antecedents and consequences among older African Americans.

### Strengths and Limitations

A significant strength of this study includes using a wealth of foundational information regarding recruitment and retention of minority groups (Kammerer et al., 2019; Liljas et al., 2017; Northridge et al., 2017; Shapiro et al., 2017) to effectively engage low-income, older African American adults in an EMA study of physical activity and sedentary behavior. Findings from this study not only point to a combination of strategies that, in conjunction, were effective in recruiting and retaining a vulnerable, understudied population, but they also suggest that low-income, older African Americans found such a study acceptable. Establishing this among low-income, older African Americans is essential to future technology-based health-related research aiming to address health disparities. Despite this, the limitations of the study should be noted. First, although the research team used individuals with extensive clinical experience including both practical knowledge and tacit experience working with older African Americans, our research team was majority White. Despite efforts to establish trust and rapport with participants, recruitment could have been further facilitated by having individuals on the research team who were of similar race and ethnic identity of the target population. Second, it is possible that by recruiting older adults from wellness centers and congregate meal sites, participants in this study may be more interested in health than the general population of older adults. Despite this, participants in the present study took a similar number of steps per day as older adults in national cohort studies (Lee et al., 2019; Tudor-Locke et al., 2013). Additionally, the average number of steps per day in this study falls within the range of steps per day recommended for older adults living with disabilities and chronic illnesses (i.e., 3,500–5,500 steps/day), but less than the recommended range of steps per day for healthy older adults (i.e., 6,000–8,500 steps/day; Tudor-Locke & Myers, 2001), all of which suggests that our sample is likely no healthier than the general population of older adults. Our recruitment methods and inclusion criteria that participants were able to read and speak English fluently may have limited our ability to recruit members of other racial and ethnic minority groups. It is unclear how the methods employed in this study would be perceived by other racial and ethnic low-income minority groups and this represents an important direction for future research.

Furthermore, our inclusion criteria also required that participants be able to see and utilize a smartphone’s basic functions, which may have resulted in a more technologically savvy sample. Despite this, 36% of participants in the present study reported not owning a smartphone. Interestingly, there was no difference in study enrollment by smartphone ownership (*p* = .36). Regarding acceptability measures, there was no difference in perceptions of the length of questionnaires or the daily demands of the study by smartphone ownership; however, perceptions of wearing the activity monitor and the demands of the study relative to the compensation differed by smartphone ownership. Older adults who did not own smartphones expressed higher levels of agreement that wearing the activity monitor was a nuisance (no smartphone: *M* = 2.5, *SD* = 1.0; smartphone: *M* = 1.9, *SD* = 0.8; *t*(53.9) = 2.5, *p* = .01) and lower levels of agreement that the demand of the study was reasonable for the compensation (no smartphone: *M* = 3.7, *SD* = 0.9; smartphone: *M* = 4.1, *SD* = 0.5; *t*(41.3) = −2.2, *p* = .03) compared to smartphone owners. There were no differences in willingness to participate in future research or an additional data collection period by smartphone ownership (*p* > .05). It may be that older African American adults who do not own a smartphone may be just as willing to participate in smartphone-based EMA research but may consider some technology-related aspects of the study more burdensome and therefore expect greater compensation compared to those who own a smartphone.

Even so, compliance with the EMA protocol in the present study was similar to compliance with a physical activity EMA study among populations of primarily White older adults in which approximately 24% did not own a smartphone (Maher et al., 2018). It is possible that providing participants with a smartphone with an active data plan could enhance compliance, as the loaned device might be utilized more by participants in between questionnaires and increase the likelihood of the phone being near participants when the EMA questionnaire is delivered. Alternatively, researchers could explore using other commercially available EMA applications that are compatible with both android and iOS platforms and, for those who have a smartphone, would allow participants to download the EMA application to their personal device. However, given documented privacy concerns of minority adults, it is unclear if downloading a research application to one’s phone would be viewed positively by all individuals (James & Harville, 2017; Liljas et al., 2017; Shapiro et al., 2017).

### Translational Implications

To effectively reduce health disparities, research relies on effective recruitment and retention of vulnerable populations often underrepresented in research. This study provided practical tips on how to effectively engage and retain vulnerable populations such as low-income, older African Americans in mobile health research, including partnering with existing programs that serve these communities, integrating staff who have experience and education in gerontology, and devoting appropriate resources to training participants on mobile health research tools. Findings from this study speak to low-income older African Americans’ willingness to participate and ability to successfully participate in mobile health research, more broadly, given appropriate training and care from the research team while enhancing the ease and accessibility of participation. From a health promotion perspective, understanding health behavior engagement in vulnerable populations at the greatest risk for chronic health conditions is a critically important step toward effective intervention development and improved health outcomes.

## Summary and Conclusions

Low-income, older African Americans reported positive perceptions of a smartphone-based EMA study of physical activity and sedentary behavior. Coupling demonstrated strategies for engaging older African American adults in health-related research studies and having experienced, culturally competent staff members and available technical support lead to effective, recruitment and retention efforts. Although this work demonstrates promise for engaging vulnerable, underrepresented populations, important questions remain about which recruitment and retention strategies are most impactful among older African Americans as well as the recruitment and retention of other vulnerable or underrepresented populations (e.g., Asian, Hispanic/Latino older adults) in mobile health research. Additionally, questions remain about the feasibility and acceptability of using intensive behavior change protocols such as Ecological Momentary Interventions within these populations.
